# Data driven inference for the repulsive exponent of the Lennard-Jones potential in molecular dynamics simulations

**DOI:** 10.1038/s41598-017-16314-4

**Published:** 2017-11-29

**Authors:** Lina Kulakova, Georgios Arampatzis, Panagiotis Angelikopoulos, Panagiotis Hadjidoukas, Costas Papadimitriou, Petros Koumoutsakos

**Affiliations:** 1 Computational Science and Engineering Laboratory, Clausiusstrasse 33, ETH Zürich, CH-8092 Switzerland; 2 Department of Mechanical Engineering, University of Thessaly, Pedion Areos, GR-38334 Volos Greece; 3 Present Address: D.E.Shaw Research LLC, New York, NY 10036 USA

## Abstract

The Lennard-Jones (LJ) potential is a cornerstone of Molecular Dynamics (MD) simulations and among the most widely used computational kernels in science. The LJ potential models atomistic attraction and repulsion with century old prescribed parameters (*q* = 6, *p* = 12, respectively), originally related by a factor of two for simplicity of calculations. We propose the inference of the repulsion exponent through Hierarchical Bayesian uncertainty quantification We use experimental data of the radial distribution function and dimer interaction energies from quantum mechanics simulations. We find that the repulsion exponent *p* ≈ 6.5 provides an excellent fit for the experimental data of liquid argon, for a range of thermodynamic conditions, as well as for saturated argon vapour. Calibration using the quantum simulation data did not provide a good fit in these cases. However, values *p* ≈ 12.7 obtained by dimer quantum simulations are preferred for the argon gas while lower values are promoted by experimental data. These results show that the proposed LJ 6-*p* potential applies to a wider range of thermodynamic conditions, than the classical LJ 6-12 potential. We suggest that calibration of the repulsive exponent in the LJ potential widens the range of applicability and accuracy of MD simulations.

## Introduction

The Lennard-Jones (LJ) potential is one of the centerpieces in Molecular Dynamics (MD) simulations, the key computational method for studying atomistic phenomena across Chemistry, Physics, Biology and Mechanics. Despite the widespread use of MD simulations, an often overlooked fact is that the classic LJ potential involves a century old and rather ad-hoc prescribed repulsion exponent. In this study we demonstrate that this parameter needs to be modified in order to enhance the predictive capabilities of MD simulations.

The structure of the LJ potential depends on the inter-atomic distance $(r)$ and consists of two parts: an attractive term −*r* ^−*q*^ that models the Van der Waals forces and a repulsive term *r* ^−*p*^ that models the Pauli repulsion. While the exponent $q\,=\,6$ has a theoretical justification^1^, the *p* = 12 exponent was chosen for computational efficiency as the square of the attractive term. In addition, two scaling parameters $\sigma$ and $\varepsilon$ control the shape of the potential. The $\varepsilon$ and $\sigma$ parameters have been the subject of numerous calibration studies^2–5^ and more recently the subject of Bayesian inference techniques^6,7^. Bayesian Uncertainty Quantification (UQ) employs experimental data and provides a probability distribution of the parameters. The parameter uncertainty can then be propagated by the model in order to obtain robust predictions on a quantity of interest^7,8^. In cases where the data sets correspond to different inputs for the system, e.g. different thermodynamic conditions, the use of Hierarchical Bayesian (HB) methods provides a stable method for UQ^9,10^.

Here we employ a HB method to infer the parameters $(\varepsilon ,\,\sigma ,\,p)$ of LJ 6-$p$ force-field. In the past, several values of the exponent $p$ of the LJ 6-$p$ potential, ranging from 10 to 20, have been considered^11^. The authors calibrated using pressure and viscosity data for various thermodynamic conditions and concluded that the exponent 12 is the best choice.

We perform  the HB inference for the LJ 6-12 and LJ 6-$p$ parameters of argon based on experimental RDFs of liquid argon and saturated argon vapor for six different temperature and pressure pairs, as well as on one dataset from quantum calculations, representing gaseous argon. We present a rigorous model selection process for the LJ 6-12 vs LJ 6-$p$ potentials for each of the cases and perform robust posterior predictions for the diffusion coefficient and density.

We find that the most likely values for the LJ repulsive exponent for liquid argon are $p\approx 6.5$, strongly differing from the value of *p* = 12 that is being used, while the gaseous argon is simulated best with the exponent $p\approx 12.7$, much closer to the conventional one. We remark that our results have been obtained in the case of a simple system. However, we consider that they offer significant evidence that the repulsive exponent should be reconsidered when the parameters of the LJ potential are being fitted to data.

## Results

In our work, we perform several inferences of the parameters of the classical and modified LJ potentials for argon at different thermodynamic conditions. Here and further, $\vartheta$ denotes a set of parameters to be calibrated.

We first calibrate the parameters $\varepsilon ,\sigma$ of the classical LJ 6-12 potential, as well as the LJ model error parameter ${\sigma }_{n}$, which accounts for the inadequacy of the LJ description, even with the best possible parameters, as compared to the physical reality. The parameter set $\vartheta$ is thus equal to $\{\varepsilon ,\sigma ,{\sigma }_{n}\}$. We use RDF as data and denote this inference as ${B}_{\mathrm{12,}R}$. Subsequently, we include the exponent $p$ of the repulsion term into the parameter set (inference ${B}_{p,R}$) and perform model selection for the LJ 6-12 and LJ 6-$p$ force fields.

We then perform a non-hierarchical and a hierarchical Bayesian inference for each of the potentials using the methodology from ref.^10^. In the non-hierarchical inference the parameters for each data set are being inferred completely independently with a common prior information, encoded in $p({\boldsymbol{\vartheta }}| {\mathcal M} )$, see Supplementary Fig. S1c. In HB inference the parameters corresponding to each data set are being connected to a hyper-parameter ${\boldsymbol{\psi }}$, see Supplementary Fig. S1b. In this approach the prior information of the model parameters is data dependent, $p({\boldsymbol{\vartheta }}|{\boldsymbol{\psi }},\overrightarrow{{\boldsymbol{d}}})$, where $\overrightarrow{{\boldsymbol{d}}}$ denotes the set of all available data, allowing information to flow between the different data sets leading to more robust and accurate predictions for the model parameters. The aim of this procedure is to infer the hyper-parameters ${\boldsymbol{\psi }}$ governing the variability of the parameters ${\boldsymbol{\vartheta }}$ between the different sets of data and to subsequently update the distributions of the parameters ${\boldsymbol{\vartheta }}$ (see Supplementary Material S1 for details). These inferences are denoted *HB*_12,*R*_ and *HB*_*p*,*R*_.

The experimentally measured RDFs are taken from ref.^12^. The RDFs are computed for 6 temperature/pressure pairs $(T,P)$. We denote the pairs as follows: ${L}_{1}=\mathrm{(84.4,}\,\mathrm{0.8)}$, ${L}_{2}=\mathrm{(91.8},\,\mathrm{1.8)}$, ${L}_{3}=\mathrm{(126.7,}\,\mathrm{18.3)}$, ${L}_{4}=\mathrm{(144.1,}\,\mathrm{37.7)}$, ${L}_{5}=\mathrm{(149.3},\mathrm{46.8)}$, $V=\mathrm{(149.3},\mathrm{43.8)}$, where $L$ stands for “liquid” and $V$ stands for “vapor”. The corresponding datasets (RDFs) are denoted as ${R}_{Li}$ for liquid and ${R}_{V}$ for vapor.

Finally, we perform the inference with LJ 6-$p$ using quantum dimer energy calculations from ref.^13^ as data, which corresponds to the gaseous argon. We then compare the obtained parameter distribution with those computed for the liquid argon and saturated argon vapour from the RDF data. The quantum dimer dataset is denoted as $Q$ and the corresponding inference is denoted as ${B}_{p,Q}$.

### Calibration of LJ 6-12

**Figure 1:**
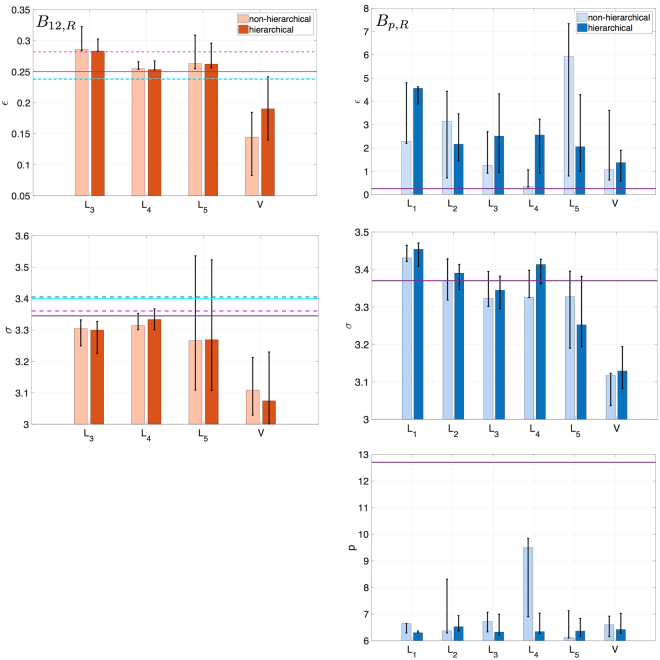
Posterior parameter values: MPVs along with 5–95% quantiles obtained in ${B}_{\mathrm{12,}R}$ (light red), $H{B}_{\mathrm{12,}R}$ (dark red), ${B}_{p,R}$ (light blue), $H{B}_{p,R}$ (dark blue). Horizontal lines for LJ 6–12 indicate the reference values: ref.^2^ (magenta dashed line), ref.^5^ (purple solid line), ref.^3^ (cyan solid line), ref.^4^ (blue dashed line). Horizontal line for LJ 6-$p$ indicates the MPV for ${B}_{p,Q}$.

We present results of parameter calibration for $\varepsilon ,\sigma ,{\sigma }_{n}$, while $p$ is fixed to 12. We use a wide enough uniform prior for each of the parameters and each of the datasets ${R}_{Li}$, ${R}_{V}$ (${\boldsymbol{\vartheta }}\in [0.05,\mathrm{3]}\times \mathrm{[3},\mathrm{4]}\times$ ${\mathrm{[10}}^{-6},1]$). We observe that the values which were obtained in the calibration process are close to those found in literature (Table 1). In Fig. 1 the most probable values (MPV) of the parameters along with 5–95% quantiles is presented (light red). Notice that results are only presented for the four out of six datasets as the LJ 6–12 potential failed to simulate the liquid argon for conditions ${L}_{1}$ and ${L}_{2}$. By “failed” we mean that there were no parameters for LJ 6-12 within the prior bounds which would produce liquid argon, the MD simulation was trapped in either gas or solid phase.

**Table 1:** LJ parameters for argon used in literature. The last row shows the data used for fitting. Notations: *T* (temperature), *P* (pressure), *ρ* (density), *B* (second virial coefficient), *E* (energy), *TP* (gas-liquid transition pressure), *L* (latent heat of evaporation).

	Ref.^3^	Ref.^2^	Ref.^4^	Ref.^5^
$\varepsilon$	0.2385	0.2824	0.2381	0.2498
$\sigma$	3.4000	3.3605	3.4050	3.3450
$T$	94.4	86.64–168.86	137.77	88–127
$\rho$	1.374	0.435–1.479	0.156, 0.972	0.283–3.897
Phase	liquid	gas, liquid, solid	gas + liquid	gas + liquid
Data	RDF	$P$, $E$	$TP$, $\rho$, $L$	$P$, $B$

**Figure 2:**
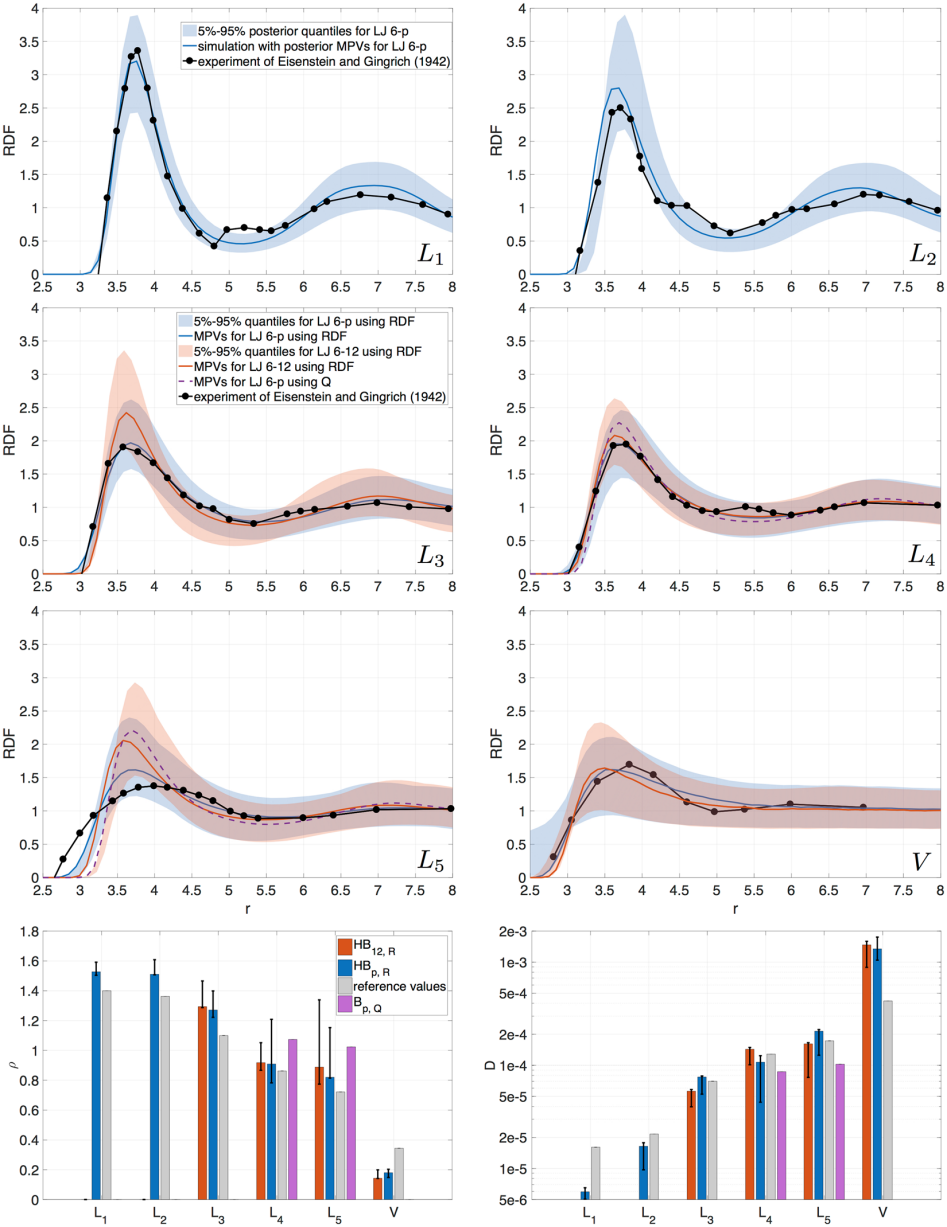
Robust posterior predictions: MPVs along with 5–95% quantiles obtained in *HB*_*p*,*R*_ (blue), *HB*_12*,R*_ (red), MPV obtained in *B*_*p*,*Q*_ (purple). Black line with dots: experimental data for RDF. Grey bars: experimental data for *ρ*, analytically computed values for *D*.

A large difference in the values of ${\varepsilon }$ for liquid and vapor is observed, which implies that one cannot perform the simulations using the same parameters for the two phases. We define the uncertainty in a parameter as the ratio of the 5–95% quantile spread to the MPV. The uncertainty in $\varepsilon$ varies from 14% to 20% depending on the dataset, while the value of $\sigma$ is identified more precisely with uncertainty of 2–6%. This difference is attributed to the type of data used in the inference process: the location of the RDF peak, which gives the most significant contribution to the sum of squared errors (SSE) in the log-likelihood, is more sensitive to $\sigma$. On the other hand, $\varepsilon$ affects the height of the RDF peak which has a smaller contribution to the log-likelihood. Despite the generally small uncertainty in $\sigma$, we observe it to be quite big for ${L}_{5}$, as compared to the other liquid cases. The reason for this lies in the shape of the RDF for ${L}_{5}$. Fig. 2 shows that the RDF for ${L}_{5}$, unlike for the other liquid cases, is very flat. The RDF peak location is controlled by $\sigma$, and since the peak is not well defined for ${L}_{5}$, the sampling algorithm allows for a wide variety of $\sigma$ values.

Next, we infer the LJ parameters using the HB approach. We select the prior $p({{\boldsymbol{\vartheta }}}_{i}|{\boldsymbol{\psi }})$ by using Bayesian model selection (see Supplementary Material S2 for details).

The values of the LJ parameters are presented in Fig. 1 (dark red). The MPVs and the quantiles of the parameters are almost the same as in the ${B}_{\mathrm{12,}R}$, which means that for each dataset ${{\boldsymbol{d}}}_{i}\in \{{R}_{L1},\ldots ,{R}_{L5},{R}_{V}\}$ no information about the parameters can be extracted from the other datasets.

The full set of the MPVs and distribution quantiles for each dataset ${R}_{Li}$, ${R}_{V}$ is given in Table 3, while the full posterior distributions are shown in Supplementary Fig. S3.

**Table 3:** Posterior values of each parameter $\vartheta \in \{\varepsilon ,\sigma ,{\sigma }_{n}\}$ of LJ 6-12: MPV $b(\vartheta )$ and 5–95% quantiles $q(\vartheta )$. The values are rounded from 16-digit precision.

		${\boldsymbol{b}}({\boldsymbol{\varepsilon }})$	${\boldsymbol{q}}({\boldsymbol{\varepsilon }})$	${\boldsymbol{b}}({\boldsymbol{\sigma }})$	${\boldsymbol{q}}({\boldsymbol{\sigma }})$	${\boldsymbol{b}}({{\boldsymbol{\sigma }}}_{{\boldsymbol{n}}})$	${\boldsymbol{q}}({{\boldsymbol{\sigma }}}_{{\boldsymbol{n}}})$
${B}_{\mathrm{12,}R}$	${L}_{3}$	0.286	[0.284, 0.323]	3.305	[3.250, 3.332]	0.168	[0.158, 0.314]
	${L}_{4}$	0.255	[0.254, 0.266]	3.314	[3.301, 3.353]	0.089	[0.080, 0.140]
	${L}_{5}$	0.263	[0.255, 0.309]	3.266	[3.110, 3.536]	0.317	[0.292, 0.586]
	$V$	0.144	[0.083, 0.184]	3.109	[3.029, 3.213]	0.147	[0.119, 0.304]
$H{B}_{\mathrm{12,}R}$	${L}_{3}$	0.283	[0.283, 0.303]	3.300	[3.226, 3.327]	0.177	[0.156, 0.310]
	${L}_{4}$	0.253	[0.254, 0.267]	3.333	[3.301, 3.367]	0.073	[0.073, 0.147]
	${L}_{5}$	0.262	[0.256, 0.296]	3.269	[3.108, 3.523]	0.337	[0.301, 0.579]
	$V$	0.190	[0.140, 0.242]	3.075	[3.001, 3.229]	0.180	[0.192, 0.385]

### Calibration of LJ 6-$p$

#### Dataset $R$

**Figure 3:**
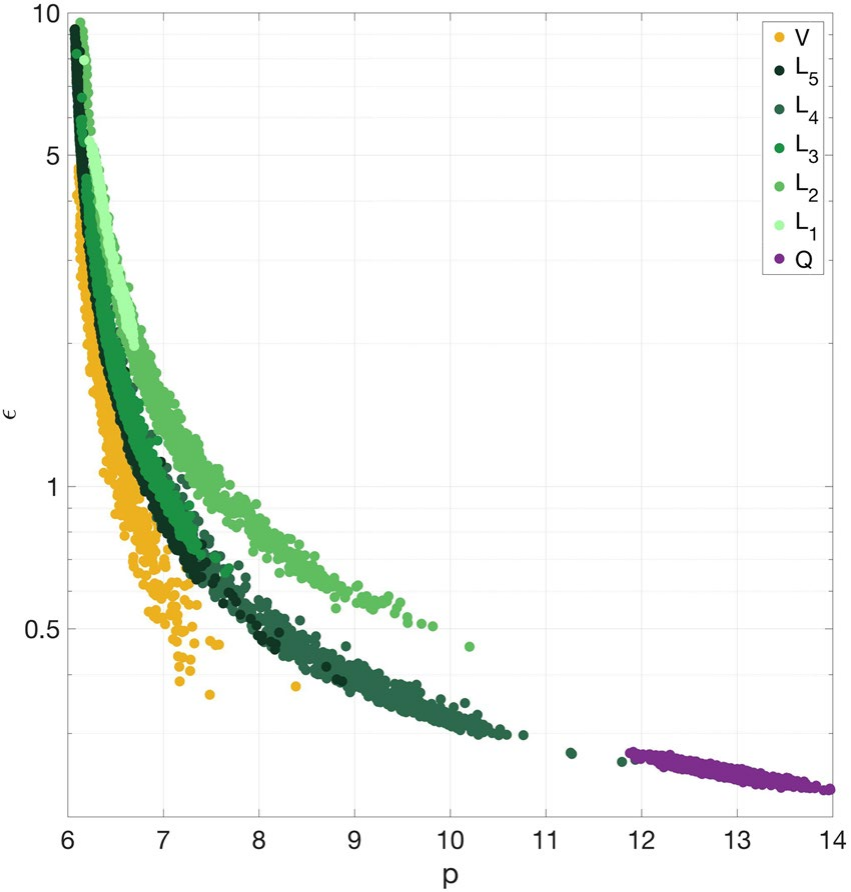
Posterior samples of *B*_*p*,*R*_ projected onto (*p*, *ε*) subspace: yellow circles correspond to *V*, green circles correspond to *L*_*i*_ in the temperature increasing order from the lightest to the darkest color, purple circles correspond to *Q*. Note that the *ε* scale is logarithmic.

Here we include the LJ exponent $p$ into the parameter set ${\boldsymbol{\vartheta }}$. As in the LJ 6-12 case, we choose a uniform prior with wide enough bounds ($\mathrm{[0.05},\mathrm{10]}\times \mathrm{[3},\mathrm{4]}\times \mathrm{[6.01},\mathrm{15]}\times {\mathrm{[10}}^{-6},\mathrm{1]}$). Note that with LJ 6-$p$ the sampling algorithm dictated much wider bounds for $\varepsilon$ compared to the LJ 6-12 case. As will be seen later, this is due to a strong correlation between $\varepsilon$ and $p$. We observe again the non-transferability of the LJ parameters from liquid to vapor simulations: the values of $\sigma$ lie in disjoint domains for ${L}_{i}$ and $V$ (Fig. 1). Being a more flexible potential, LJ 6-$p$ can simulate a wider range of thermodynamic conditions, including ${L}_{1}$ and ${L}_{2}$, which result in the values of LJ parameters similar to those obtained for the other three liquid conditions. We observe that the 95% quantile of $p$, as well as its MPV, is for four out of six RDF datasets below $7.5$ and for all the datasets below 10, which is much smaller than the conventional 12. This can be explained by the fact that the repulsion energies predicted by the standard 6-12 LJ potential are very high for the liquids. The configurations with such energies happen with probability close to zero, and the MD simulation is not able to sample them. As in the LJ 6-12 case, the parameter $\varepsilon$ exhibits significant variation within each dataset ${R}_{Li}$, ${R}_{V}$ (uncertainty 110–216%, computed the same way as for LJ 6-12), while $p$ and $\sigma$ are well-defined with the uncertainty of 5–30% and 1–6%, respectively. In addition, $\varepsilon$ differs substantially among the RDF datasets, but always in accordance with $p$: the higher the $p$, the lower the $\varepsilon$ (see Fig. 3). We observe that the uncertainty in $\sigma$ is once again bigger for ${L}_{5}$, as compared to the other liquid cases, which is consistent with the LJ 6-12 inference.

Similarly with the inference of the LJ 6-12 parameters, we proceed by calibrating the parameters using the HB approach. Details for the selection of the prior can be found in Supplementary Material S2. The results of the inference are given in Fig. 1. We observe that the uncertainty in $\varepsilon$ gets significantly reduced for conditions ${L}_{1}$, ${L}_{2}$, ${L}_{5}$ and $V$ indicating that the inference benefited from the information contained in the two remaining datasets ${R}_{L3}$ and ${R}_{L4}$ with narrow posterior distributions of $\varepsilon$ (Supplementary Figs S2 and S4). On the other hand, the uncertainty in $\varepsilon$ for ${L}_{3}$ and ${L}_{4}$ increases adjusting to the wide ranges in the other four cases. A similar situation can be seen for $p$, where narrow distributions for ${L}_{1}$, ${L}_{3}$, ${L}_{5}$, $V$ shift the posterior values for ${L}_{2}$ and ${L}_{4}$. The RDF is, as noticed before, very sensitive to the changes in $\sigma$, which controls the location of the LJ potential well, and therefore $\sigma$ is well determined for each of the datasets ${R}_{{L}_{i}}$, ${R}_{V}$ and extracts almost no information from the other data sets.

#### Dataset $Q$

We examine the suitability of the repulsion exponent 12 by performing a calibration using as data the calculated quantum dimer scans of argon. These data describe the behavior of the gaseous argon. We infer the LJ 6-$p$ parameters by fitting the LJ potential to the binding energy of the quantum dimer (Fig. 1). The resulting value of $p$ is much closer to the conventional 12 (Table 4), suggesting that for the gaseous argon, unlike for the liquid one, LJ 6-12 is a reasonable choice.

**Table 4:** Posterior values of each parameter $\vartheta \in \{\varepsilon ,\sigma ,p,{\sigma }_{n}\}$ of LJ 6-$p$: MPV $b(\vartheta )$ and 5–95% quantiles $q(\vartheta )$. The values are rounded from 16-digit precision.

		${\boldsymbol{b}}({\boldsymbol{\varepsilon }})$	${\boldsymbol{q}}({\boldsymbol{\varepsilon }})$	${\boldsymbol{b}}({\boldsymbol{\sigma }})$	${\boldsymbol{q}}({\boldsymbol{\sigma }})$	${\boldsymbol{b}}({\boldsymbol{p}})$	${\boldsymbol{q}}({\boldsymbol{p}})$	${\boldsymbol{b}}({{\boldsymbol{\sigma }}}_{n})$	${\boldsymbol{q}}({{\boldsymbol{\sigma }}}_{{\boldsymbol{n}}})$
${B}_{p,R}$	${L}_{1}$	2.286	[2.193, 4.794]	3.431	[3.422, 3.464]	6.644	[6.292, 6.647]	0.157	[0.185, 0.302]
	${L}_{2}$	3.134	[0.712, 4.416]	3.369	[3.319, 3.428]	6.370	[6.293, 8.313]	0.222	[0.215, 0.518]
	${L}_{3}$	1.250	[0.914, 2.700]	3.322	[3.301, 3.395]	6.715	[6.332, 7.068]	0.098	[0.080, 0.159]
	${L}_{4}$	0.337	[0.322, 1.060]	3.325	[3.325, 3.398]	9.501	[6.900, 9.842]	0.057	[0.066, 0.153]
	${L}_{5}$	5.928	[0.794, 7.318]	3.328	[3.190, 3.395]	6.116	[6.102, 7.126]	0.164	[0.168, 0.326]
	$V$	1.065	[0.625, 3.611]	3.117	[3.037, 3.122]	6.604	[6.151, 6.923]	0.100	[0.099, 0.190]
$H{B}_{p,R}$	${L}_{1}$	4.561	[3.890, 4.626]	3.454	[3.408, 3.471]	6.302	[6.296, 6.366]	0.422	[0.450, 0.602]
	${L}_{2}$	2.081	[1.333, 3.515]	3.387	[3.335, 3.424]	6.565	[6.340, 7.051]	0.211	[0.178, 0.351]
	${L}_{3}$	2.506	[0.941, 4.325]	3.345	[3.295, 3.382]	6.324	[6.198, 6.996]	0.093	[0.081, 0.186]
	${L}_{4}$	2.588	[0.892, 3.676]	3.403	[3.358, 3.432]	6.339	[6.226, 7.063]	0.082	[0.075, 0.146]
	${L}_{5}$	2.055	[0.992, 4.281]	3.252	[3.194, 3.382]	6.364	[6.169, 6.837]	0.183	[0.159, 0.316]
	$V$	1.371	[0.582, 1.896]	3.129	[3.082, 3.194]	6.422	[6.277, 7.025]	0.111	[0.103, 0.233]
${B}_{p,Q}$		0.252	[0.239, 0.261]	3.370	[3.367, 3.375]	12.703	[12.333, 13.309]	0.006	[0.006, 0.010]

The full set of MPVs and distribution quantiles of the LJ parameters for ${R}_{Li}$, ${R}_{V}$ and $Q$ is given in Table 4 and the full posterior distributions are plotted in Supplementary Figs S2, S4 and S5.

### LJ 6-12 vs LJ 6-$p$: Comparison

#### Model selection

We choose between the LJ 6-12 and LJ 6-$p$ potentials by applying the Bayes selection criterion. We observe that LJ 6-$p$ is significantly better than the LJ 6-12 for ${L}_{3}$ and ${L}_{5}$ (Table 5). Recalling that LJ 6-12 is not able to produce a liquid for ${L}_{1}$ and ${L}_{2}$, we conclude that LJ 6-$p$ is preferred for four RDF datasets out of six. In the case of ${L}_{4}$ the two potentials provide results that are indistinguishable by the Bayesian model selection. The only dataset on which the LJ 6-12 potential produces better results (3 times more probable than LJ 6-$p$) is $V$, the vapor case. That brings us to the conclusion that LJ 6-$p$ is either much better or not worse than LJ 6-12 for all the liquid cases considered. For the vapor case, the LJ 6-$p$ is over parametrized, as compared to LJ 6-12.

**Table 5:** Log-evidences ${E}_{\mathrm{12,}R}$ (${E}_{p,R}$) for ${B}_{\mathrm{12,}R}$ (${B}_{p,R}$).

	${{\boldsymbol{E}}}_{{\bf{12}},{\boldsymbol{R}}}$	${{\boldsymbol{E}}}_{{\boldsymbol{p}},{\boldsymbol{R}}}$	${{\boldsymbol{e}}}^{{{\boldsymbol{E}}}_{{\boldsymbol{p}},{\boldsymbol{R}}}{\boldsymbol{-}}{{\boldsymbol{E}}}_{{\bf{12}},{\boldsymbol{R}}}}$
${L}_{1}$	—	−7.05	—
${L}_{2}$	—	−14.8	—
${L}_{3}$	−9.72	2.81	2.74 × 10^5^
${L}_{4}$	5.10	5.18	1.09
${L}_{5}$	−15.8	−8.76	1.18 × 10^3^
$V$	−3.83	−4.94	3.31 × 10^−1^

#### LJ potentials

**Figure 4:**
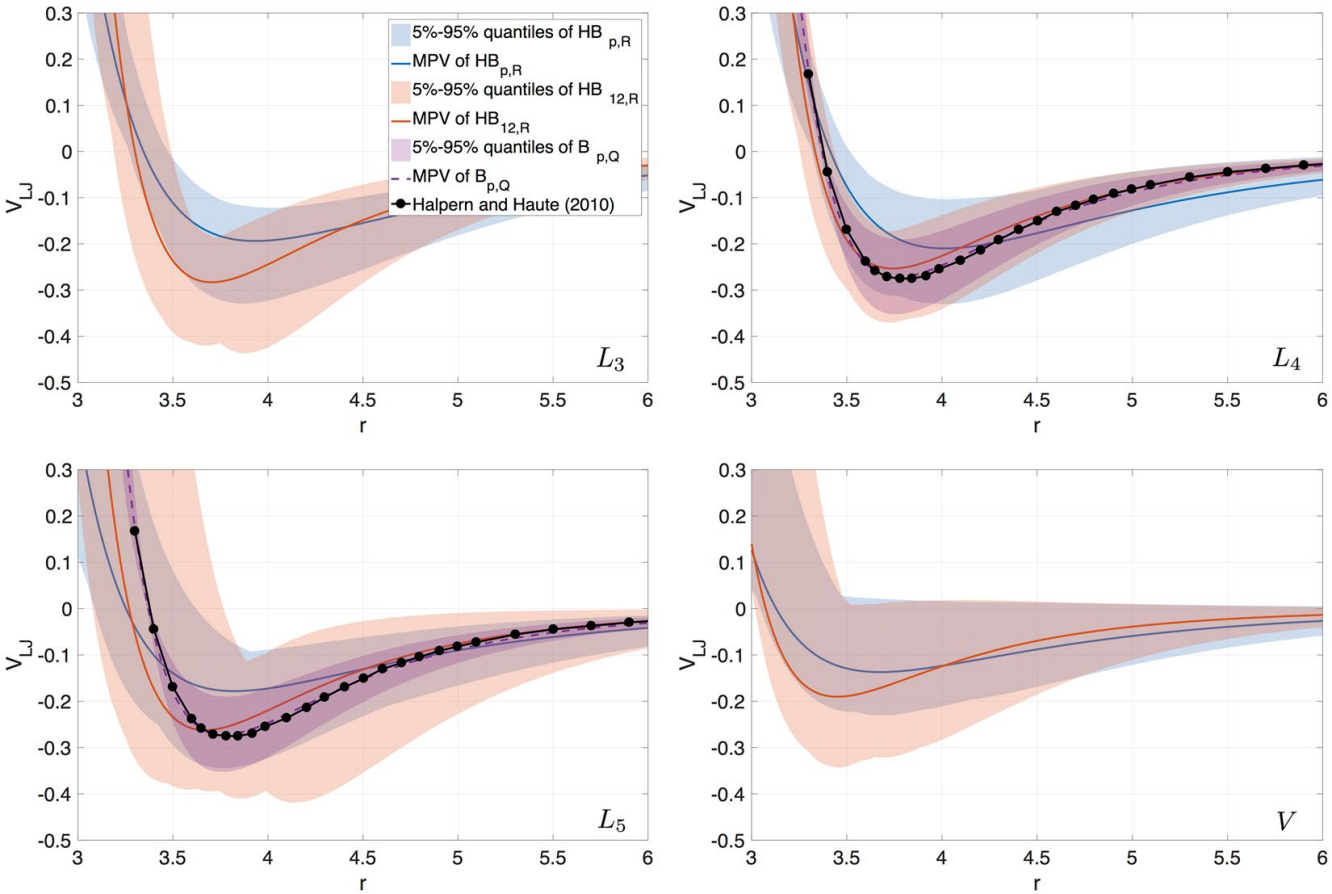
Posterior LJ potentials comparison: MPVs along with 5–95% quantiles obtained in *HB*_*p*,*R*_ (blue), *HB*_12,*R*_ (red), *B*_*p*,*Q*_ (purple). Black line with dots: quantum dimer calculations.

Studying the reasons for LJ 6-$p$ being more plausible than LJ 6-12, we take a closer look at the inferred shapes of the potentials. We observe a very stable correlation in the $(p,\varepsilon )$ subspace (Fig. 3) for all the datasets used. This result is expected as $p$ regulates the strength of the repulsion and $\varepsilon$ alters the strength of both repulsion and attraction simultaneously. The difference between the ${R}_{i}$ and $Q$ datasets shows up in the region of the subspace which gets populated. The quantum dimer-based calibration prefers high values of $p$, which correspond to the tails of the distributions inferred using the RDF data. We performed a calibration with ${L}_{3}$ and narrow prior bounds ($p\in \mathrm{[12},\mathrm{14]}$) to see whether this is indeed a tail of the full posterior distribution (Supplementary Fig. S6). The narrow posterior values are below $3.95$, while the values of the full posterior start from $4.18$, which explains why the tails of the full distributions for ${L}_{i}$ and $V$ have a negligible number of samples in the region $p\in \mathrm{[12,14]}$ preferred by the $Q$-based inference. As the parameters $\varepsilon$ and $p$ are highly correlated, one could expect that the inference will be able to recover values of $\varepsilon$ for LJ 6-12 such that the resulting potential is close to the inferred LJ 6-$p$. However, the effect that $p$ and $\varepsilon$ have on the LJ potential is not entirely the same. As $\varepsilon$ acts as a scaling factor for the whole potential, it is not able to make the potential less deep and at the same time flat enough to avoid switching to the gas phase (compare simulations with MPVs for ${L}_{5}$, $V$ in Fig. 4). The same reasoning can be applied to explain the inability of LJ 6-12 to drive ${L}_{1}$ and ${L}_{2}$ to the liquid phase: the potential is too repulsive, frustrating the liquid packing, and the system behaves either like a gas or like a solid (note that ${L}_{1}$ is close to the argon triple point). The full set of the inferred LJ 6-12 and LJ 6-$p$ potentials is given in Fig. 4.

#### Robust posterior prediction

The quality of the pred ictions, made for a quantity of interest (QoI) different than the one used for the inference, quantifies the predictive power of the model (see Supplementary Material S1.2). We obtain robust predictions of the RDF, density *ρ* and diffusion coefficient *D* of argon by propagating the posterior LJ parameters uncertainty into these quantities.

We measure the error Δ*g* of the prediction of the scalar quantity $g$ as

1 $${\rm{\Delta }}g=\frac{1}{N}\sum _{k=1}^{N}{(\frac{{g}_{k}-{r}_{k}}{{r}_{k}})}^{2}\,,$$

where *N* ≤ 6 is the number of thermodynamic conditions for which the prediction can be made, ${g}_{k}$ is the prediction made using the MPV and ${r}_{k}$ is the reference value. The reference values for $\rho$ are experimental measurements taken from ref.^12^. The reference values for $D$ are computed analytically using the equations from ref.^14^. The accuracy of the fit for these computations is 0.7%. The error of the RDF is computed as an average over all the thermodynamic conditions of the mean squared error between the computed and the experimental RDF. The predictions are compared on three different sets of conditions: (1) the conditions which can be simulated using MPVs obtained in all the three inferences $H{B}_{\mathrm{12,}R}$, $H{B}_{p,R}$, and ${B}_{p,Q}$ (${L}_{4}$, ${L}_{5}$), (2) the conditions which can be simulated using MPVs obtained in the inferences $H{B}_{\mathrm{12,}R}$ and $H{B}_{p,R}$ (${L}_{3}-{L}_{5}$, $V$), (3) the conditions which can be simulated using MPVs obtained in the inference $H{B}_{p,R}$ (${L}_{1}-{L}_{5}$, $V$).

The predictions made using the results of $H{B}_{p,R}$ are the most accurate for all the QoIs considered and for all but one case where $H{B}_{\mathrm{12,}R}$ gives a better result (see Table 6). On the other hand, the predictions made using the results of ${B}_{p,Q}$ are the least accurate for all the QoIs. Additionally, the inferences $H{B}_{\mathrm{12,}R}$ and ${B}_{p,Q}$ result in LJ potentials which cannot be used to simulate all the thermodynamic conditions. We conclude that the $H{B}_{p,R}$ produces a better LJ model than $H{B}_{\mathrm{12,}R}$, and that ${B}_{p,Q}$ does not result in a good model for liquid argon or saturated argon vapor.

**Table 6:** Errors of robust posterior predictions of RDF, density and diffusion coefficient using LJ 6–12 and LJ 6-$p$. We denote ${S}_{1}=\{{L}_{4},{L}_{5}\}$ (all inferences produce the correct argon phase), ${S}_{2}=\{{L}_{3}-{L}_{5},V\}$ (${B}_{p,Q}$ produces wrong phase), ${S}_{3}=\{{L}_{1}-{L}_{5},V\}$ (${B}_{p,Q}$ and $H{B}_{\mathrm{12,}R}$ produce wrong phase).

	**Δ RDF**			${\boldsymbol{\Delta }}{\boldsymbol{\rho }}$			${\boldsymbol{\Delta }}{\boldsymbol{D}}$		
	${{\boldsymbol{S}}}_{{\bf{1}}}$	${{\boldsymbol{S}}}_{{\bf{2}}}$	${{\boldsymbol{S}}}_{{\bf{3}}}$	${{\boldsymbol{S}}}_{{\bf{1}}}$	${{\boldsymbol{S}}}_{{\bf{2}}}$	${{\boldsymbol{S}}}_{{\bf{3}}}$	${{\boldsymbol{S}}}_{{\bf{1}}}$	${{\boldsymbol{S}}}_{{\bf{2}}}$	${{\boldsymbol{S}}}_{{\bf{3}}}$
${B}_{p,Q}$	0.087	—	—	0.118	—	—	0.136	—	—
$H{B}_{\mathrm{12,}R}$	0.071	0.050	—	0.029	0.108	—	0.009	1.573	—
$H{B}_{p,R}$	0.016	0.024	0.027	0.011	0.068	0.049	0.043	1.230	0.898

We note that the values of $D$ differ by an order of magnitude for liquid and vapor which explains the huge deterioration of the predictions on the sets of conditions that include $V$.

The MPVs of $D$ and $\rho$ along with the corresponding quantiles are presented in Fig. 2. The same values for RDF are given in Fig. 2.

## Discussion

We have performed a systematic study of the modified 6-$p$ Lennard-Jones potential for liquid and gaseous argon using Hierarchical Bayesian inference with data from experiments and quantum mechanics simulations. Our results show that the value *p* = 12 of the repulsive exponent needs to be calibrated together with the other LJ parameters. In the case of liquid argon we obtain much better predictions with a smaller value $p\approx 6.5$, while for the gaseous argon the classical $p=12$ or a slightly bigger $p\approx 12.7$ results in a better agreement with the data.

Our results contradict the conclusion of ref.^11^, where LJ potentials with $p$ ranging from 10 to 20 with a step 2 were fit to viscosity and pressure data, and the potential with $p=12$ showed better predictions for different thermodynamic conditions. Even though the conclusion of ref.^11^ formally contradicts our findings, we still consider it a good match, for the following three reasons. Firstly, in ref.^11^, the average errors of pressure and viscosity predictions for argon strictly increase with the increase of the exponent from 12 to 20, which is consistent with our results (in the sense that exponents smaller than 14 are preferred by all our inferences). Secondly, for the exponents $p=10$ and $p=12$ the authors of ref.^11^ report quite close errors of approximately 2% and 4%, respectively. Finally, different data was used in the calibration process, which may, at least partially, account for the observed difference.

Taking into account the differences in $\varepsilon$ and $\sigma$ (Fig. 1), as well as in $p$, we conclude that one cannot use the same values of the LJ parameters for liquid, saturated vapor and gas. This issue is attributed to the simplicity of the LJ description: the potential, and thus the underlying physical model of the intermolecular interactions is not flexible or accurate enough to allow for the transferability of the same parameters between different physical environments. The model can be potentially improved by including more complex physics in it, such as many-body interactions with geometrical considerations or quantum corrections. For argon however, the quantum corrections are not expected to be significant^15^. At the same time, including the triple-dipole interactions was found to provide better results than the LJ 6-12 for all thermodynamic states of argon^2^. We emphasize however that ab-initio derived forcefields including multi-body terms have also suffered from non perfect transferability of their parameters between gaseous and condensed phase environments. AMOEBA^16,17^ has been an example of a hybrid force-field that includes additional physics, and it was also calibrated using both quantum and experimental data. In the limit of a perfect model, perfect transferability should be anticipated between all physical environments. However, since all force field models are approximations to reality and QM interaction energies even at the Coupled Cluster Stochastic Dynamics (CCSD(T)) level are uncertain, we expect the same lack of transferability for their optimal parameters between different physical environments.

Smaller values of the repulsion exponent ($p\in \mathrm{(6},\mathrm{9)}$) in the Lennard-Jones potential provide better predictions for RDF, density and diffusion data (Fig. 2) than the conventional $p=12$. Additionally, these new LJ exponents allow to simulate a larger variety of thermodynamic conditions, as compared to the classical 12.

We have also examined whether the smaller exponent allows for bigger time steps in MD simulations. However, it appears that the exponent is not a critical factor for the stability of the system. that is, the energy conservation and the RDF were unaffected by the increase of the time step from 1 fs to approximately 16 fs for both LJ 6-12 and LJ 6-6.5. Further increase of the time step lead to the crash of both MD simulations. We observed similar execution times for the simulations with MPVs of LJ 6-12 and LJ 6-$p$.

From the computational point of view, usage of low computational cost interpolation-based models replacing the real simulations (called surrogates) resulted in a speed-up of 28%.

## Methods

### Molecular Dynamics

We perform MD simulations of argon using LAMMPS package^18^. The argon atoms are modeled as spheres which interact with LJ 6-$p$ potential:

2 $${V}_{LJ}(r;\,\varepsilon ,\,\sigma ,\,p)\,=\,4\varepsilon ({(\frac{\sigma }{r})}^{p}-{(\frac{\sigma }{r})}^{6})\,,$$

where $r$ is the distance between the interacting atoms and $p$ is the repulsion exponent usually taken to be 12. The parameters $\varepsilon$, $\sigma$ and $p$ are to be chosen according to the available measurements. As the Lennard-Jones interactions quickly decay with the distance, an additional computational cut-off parameter ${r}_{c}$ is usually introduced, after which the values of the potential are defined in a different fashion. As was shown in ref.^7^, the values of the cut-off are connected with the values of $\varepsilon$ and $\sigma$. However, in our work we would like to focus on the effect of the repulsion exponent, and thus leave the value of the cut-off unchanged. We assign ${r}_{c}=3\sigma$ and set the LJ values for $r > {r}_{c}$ to 0. The thermodynamic state of the system is defined by the temperature and the pressure of the argon atoms. We ensure that argon is in the liquid/vapor state by checking the self-diffusion coefficient and the density. The simulation starts with energy minimization followed by $5\times {10}^{6}$ steps, of 2 fs, in an NPT ensemble. Then the RDF is computed in the production run consisting of ${10}^{5}$ NVE integration steps of 2 fs each. The boundary conditions are periodic in each direction, the domain contains 666 argon atoms. The self-diffusion coefficient is calculated via the mean-squared displacement of the atoms, the RDF is discretized using 100 bins. The units used in the current work are given in Table 2.

**Table 2:** Units used in this work.

Name	Symbol	Unit
Temperature	$T$	K
Pressure	$P$	atm
Distance	$r$	Å
LJ well depth	$\varepsilon$	kcal/mol
LJ well location	$\sigma$	Å
LJ repulsion exponent	$p$	—
RDF model error	${\sigma }_{n}$	—
Density	$\rho$	g/cm^3^
Diffusion coefficient	$D$	cm^2^/s

### Bayesian Uncertainty Quantification

This section presents a brief description of the Bayesian inference theory. The details are given in Supplementary Material S1. Here and further in the text, small bold letters represent vectors while big bold letters represent matrices. Each random variable ***ξ*** is assumed to be continuous with a probability density function (PDF) denoted as ***ξ***.

Let $f({\boldsymbol{x}};{\boldsymbol{\vartheta }})\in {{\mathbb{R}}}^{M}$ denote the output, or a QoI, of a computational model with input ${\boldsymbol{x}}\in {{\mathbb{R}}}^{{N}_{x}}$ and parameters ${\boldsymbol{\vartheta }}=({{\vartheta }}_{1},\ldots ,{{\vartheta }}_{{N}_{{\vartheta }}})\in {{\mathbb{R}}}^{{N}_{{\vartheta }}}$. Let also ${\boldsymbol{d}}\in {{\mathbb{R}}}^{{N}_{d}}$ be a vector of experimental data corresponding to the QoI $f$ and input parameters ${\bf{x}}$. The experimental data are linked with the computational model through the likelihood function, ${\boldsymbol{d}}[{\vartheta },\,{\boldsymbol{x}}]$. A usual model assumption for the likelihood function involves a Gaussian,

3 $$p({\boldsymbol{d}}|{\boldsymbol{\vartheta }},{\boldsymbol{x}})={\mathscr{N}}(d{\boldsymbol{|}}f({\boldsymbol{x}};{\boldsymbol{\vartheta }}),{\boldsymbol{\Sigma }}),$$

where ${\boldsymbol{\Sigma }}$ is a covariance matrix that may be a function of ${\boldsymbol{\vartheta }}$. To simplify the notations, the conditioning on ***x*** is omitted below. Prior information on the parameters ${\boldsymbol{\vartheta }}$ is encoded into the probability distribution with PDF ${\boldsymbol{\vartheta }}[ {\mathcal M} ]$. We assume ${\boldsymbol{\Sigma }}={\sigma }_{n}^{2}{\boldsymbol{I}}$, where ${\boldsymbol{I}}$ is the identity matrix in ${{\mathbb{R}}}^{{N}_{{\vartheta }}\times {N}_{{\vartheta }}}$ and ${\sigma }_{n}\in {\mathbb{R}}$ is *a priori* unknown. In this work, we infer the parameters of the LJ potential together with the parameter of the covariance matrix: ${\boldsymbol{\vartheta }}=(\varepsilon ,\sigma ,{\sigma }_{n})$ or ${\boldsymbol{\vartheta }}=(\varepsilon ,\sigma ,p,{\sigma }_{n})$ depending on whether the exponent $p$ is being inferred or not.

Bayes’ theorem provides a tool for the inference of the parameters ${\boldsymbol{\vartheta }}$ conditioned on the observations ${\boldsymbol{d}}$,

4 $$p({\boldsymbol{\vartheta }}|{\boldsymbol{d}}{\boldsymbol{,}} {\mathcal M} )=\frac{p({\boldsymbol{d}}|{\boldsymbol{\vartheta }}, {\mathcal M} )p({\boldsymbol{\vartheta }}| {\mathcal M} )}{p({\boldsymbol{d}}| {\mathcal M} )}\,,$$

where $p({\boldsymbol{d}}| {\mathcal M} )=\int \,p({\boldsymbol{d}}|{\boldsymbol{\vartheta }}, {\mathcal M} )p({\boldsymbol{\vartheta }}| {\mathcal M} )d{\boldsymbol{\vartheta }}$ is a normalization constant and ${\mathcal M}$ stands for “model”, which is a set of the assumptions regarding the likelihood and the prior. We remark that the denominator $p({\boldsymbol{d}}| {\mathcal M} )$, called model evidence, is used for model selection (see Supplementary Material S1.3).

In certain cases the data may correspond to different input variables ${\bf{x}}$ of the model, one of the examples is pressure and temperature used in this work. Let $\overrightarrow{{\boldsymbol{d}}}=\{{{\boldsymbol{d}}}_{1},\ldots ,{{\boldsymbol{d}}}_{N}\}$ be the set of all provided data with ${{\boldsymbol{d}}}_{i}\in {{\mathbb{R}}}^{{N}_{{\vartheta }_{i}}}$, where each ${{\boldsymbol{d}}}_{i}$ corresponds to different input ${{\boldsymbol{x}}}_{i}$. In this case one wishes to infer different parameters, ${{\boldsymbol{\vartheta }}}_{i}\in {{\mathbb{R}}}^{{N}_{\vartheta }}$, for each dataset ${{\boldsymbol{d}}}_{i}$. Here, we assume that the parameters ${{\boldsymbol{\vartheta }}}_{i}$ depend on hyper-parameters ${\boldsymbol{\psi }}\in {{\mathbb{R}}}^{{N}_{\psi }}$, which encode the variability of ${{\boldsymbol{\vartheta }}}_{i}$ between the datasets and should also be inferred.

For the sampling of the distributions we use the Transitional Markov Chain Monte Carlo (TMCMC) algorithm^19^ (see Supplementary Material S1.1). We perform all the inferences using the open-source library ${\rm{\Pi }}$ 4U^8^ on Brutus cluster of the ETH Zurich and Piz Daint cluster of the Swiss National Supercomputing Center (CSCS). We use 2000 samples per TMCMC stage for LJ 6-12 and 4000 samples per stage for LJ 6-$p$. The parallelisation is made with MPI and internal worker threads of the ${\rm{\Pi }}$ 4U library. The task-based parallelism and the load balancing mechanisms of ${\rm{\Pi }}$ 4U provide the necessary flexibility for running MD simulations with very different execution time within TMCMC.

In order to reduce the computational cost of the simulations, we apply kriging surrogates following the methodology proposed in ref.^20^. Namely, for each Markov chain leader we build a kriging interpolating surface using the samples from the leader’s bounding box. We select the size of the box to be equal to a quarter of the current domain. The surrogate value is rejected if the kriging error is greater than 5% of the predicted value. In addition, we do not allow the kriging predictions which are outside the 5–95% quantile range of all the values obtained from MD simulations.

### Availability of materials and data

We use an open-source framework $\Pi$ 4U available at http://www.cse-lab.ethz.ch/software/Pi4U. The data we used come from refs^12,13^.

## Electronic supplementary material

Supplementary Material
