# Supplementary material for: Data driven inference for the repulsive exponent of the Lennard-Jones potential in molecular dynamics simulations
Source: Sci Rep. 2017 Nov 29;7:16576. doi: 10.1038/s41598-017-16314-4 (PMC5707428; doi:10.1038/s41598-017-16314-4)
Supplement: Supplementary file 1 — Supplementary Material [file 41598_2017_16314_MOESM1_ESM.pdf]

Supplementary Material:
Data driven inference for the repulsive exponent of the Lennard-Jones potential in molecular dynamics simulations

Lina Kulakova[1], Georgios Arampatzis[1], Panagiotis Angelikopoulos[1,2], Panagiotis Hadjidoukas[1], Costas Papadimitriou[3], and Petros Koumoutsakos[1,*]

[1]Computational Science and Engineering Laboratory, Clausiusstrasse 33, ETH Zrich, CH-8092, Switzerland
[2]Department of Mechanical Engineering, University of Thessaly, Pedion Areos, GR-38334 Volos, Greece
[3]Currently at D.E.Shaw Research LLC, 10016 NY USA
[*]petros@ethz.ch

S1 Uncertainty quantification

This section provides a detailed description of the UQ theory used in the current work.

S1.1 Sampling the posterior distribution

For the posterior distribution $p(\boldsymbol{\vartheta}\,|\,\boldsymbol{d},\mathcal{M})$ which is known up to a normalizing constant $p(\boldsymbol{d}\,|\,\mathcal{M})$, available Markov Chain Monte Carlo (MCMC) methods can be used to efficiently generate samples that quantify the uncertainty in $\boldsymbol{\vartheta}$ [1, 2, 3, 4, 5]. In our work we use the Transitional Markov Chain Monte Carlo (TMCMC) algorithm [6] with a slight modification on the MCMC proposal covariance used to overcome low acceptance rates in several runs of TMCMC which we observed. Instead of setting the covariance matrix to the scaled sample covariance of the previous generation, we use the landscape around the chain leaders to construct local covariance matrices. The sampling algorithm automatically tries to increase the radius of the neighborhood starting from 10% of the domain size until the local covariance matrix is positive definite.

S1.2 Robust posterior prediction

The uncertainty in the model parameters can be further propagated to the uncertainty in the QoI $\boldsymbol{y}$ produced by $f(\boldsymbol{x};\boldsymbol{\vartheta})$. Under the assumption on the likelihood function

$$p(\boldsymbol{d}\,|\,\boldsymbol{\vartheta},\boldsymbol{x}) = \mathcal{N}(\boldsymbol{d}\,|\,f(\boldsymbol{x};\boldsymbol{\vartheta}),\boldsymbol{\Sigma})\,, \tag{S1}$$

the probability of the model prediction conditioned on the parameters $\boldsymbol{\vartheta}$ is given by $p(\boldsymbol{y}\,|\,\boldsymbol{\vartheta},\mathcal{M}) = \mathcal{N}(\boldsymbol{y}\,|\,f(\boldsymbol{x};\boldsymbol{\vartheta}),\boldsymbol{\Sigma})$. The probability of the model prediction conditioned on the observations $\boldsymbol{d}$ is known as the robust posterior prediction and is given by [7, 8]:

$$p(\boldsymbol{y}\,|\,\boldsymbol{d},\mathcal{M}) = \int p(\boldsymbol{y},\boldsymbol{\vartheta}\,|\,\boldsymbol{d},\mathcal{M})\,d\boldsymbol{\vartheta} = \int p(\boldsymbol{y}\,|\,\boldsymbol{\vartheta},\mathcal{M})\,p(\boldsymbol{\vartheta}\,|\,\boldsymbol{d},\mathcal{M})\,d\boldsymbol{\vartheta} \approx \frac{1}{N_s}\sum_{k=1}^{N_s} p(\boldsymbol{y}\,|\,\boldsymbol{\vartheta}^{(k)},\mathcal{M})\,, \tag{S2}$$

where $\boldsymbol{\vartheta}^{(k)} \sim p(\boldsymbol{\vartheta}\,|\,\boldsymbol{d},\mathcal{M})$ and N_s is sufficiently large.

When a new QoI $\boldsymbol{z}$ produced by $g(\boldsymbol{x};\boldsymbol{\vartheta})$ is considered, $\boldsymbol{\Sigma}$ is unknown and only the parametric uncertainty is propagated into $g(\boldsymbol{x};\boldsymbol{\vartheta})$. Namely, one should estimate the density of $\sum_{k=1}^{N_s} g(\boldsymbol{x};\boldsymbol{\vartheta}^{(k)})$, where $\boldsymbol{\vartheta}^{(k)} \sim p(\boldsymbol{\vartheta}\,|\,\boldsymbol{d},\mathcal{M})$ and N_s is sufficiently large.

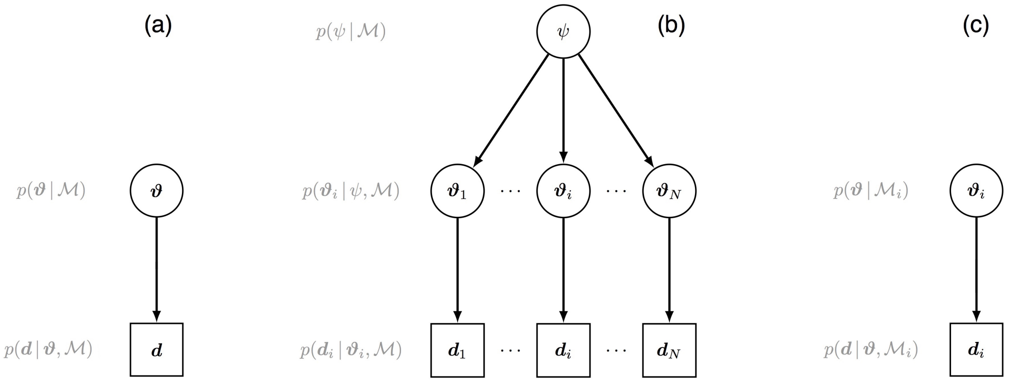

Figure S1: Bayesian networks: (a) simple network, (b) HB network, (c) network for the i-th dataset.

S1.3 Model selection

The Bayesian framework allows one to select the probabilistic model which best fits the data. The criterion for the model selection comes from the Bayes' theorem, which computes the probability of a probabilistic model $\mathcal{M}$ as

$$p(\mathcal{M} \,|\, \boldsymbol{d}) = \frac{p(\boldsymbol{d} \,|\, \mathcal{M})\, p(\mathcal{M})}{p(\boldsymbol{d})}\,, \tag{S3}$$

where $p(\mathcal{M})$ is the prior PDF of the model $\mathcal{M}_i$ and the evidence $p(\boldsymbol{d} \,|\, \mathcal{M})$ of model $\mathcal{M}$ is computed as a by-product of TMCMC.

S1.4 Hierarchical Bayesian models

In our work we follow the methodology developed in [9]. We assume that data comes split in N different datasets: $\vec{\boldsymbol{d}} = \{\boldsymbol{d}_1, \ldots, \boldsymbol{d}_N\}$ and the likelihood in the probabilistic model $\mathcal{M}_i$ is $\mathcal{N}(\boldsymbol{d}_i \,|\, f(\boldsymbol{x}; \boldsymbol{\vartheta}_i), \boldsymbol{\Sigma}_i)$. We assume that the probability of $\boldsymbol{\vartheta}_i$ depends on a hyper-parameter $\boldsymbol{\psi} \in \mathbb{R}^{N_\psi}$ and is given by a PDF $p(\boldsymbol{\vartheta} \,|\, \boldsymbol{\psi}, \mathcal{M})$, where $\mathcal{M}$ corresponds to the graph describing the relations between $\boldsymbol{\psi}$, $\boldsymbol{\vartheta}_i$ and $\boldsymbol{d}_i$, see Figure S1. Our goal is to obtain samples from the posterior distribution, $p(\boldsymbol{\vartheta}_i \,|\, \vec{\boldsymbol{d}}, \mathcal{M})$, where $\vec{\boldsymbol{d}} = \{\boldsymbol{d}_1, \ldots, \boldsymbol{d}_N\}$:

$$p(\boldsymbol{\vartheta}_i \,|\, \vec{\boldsymbol{d}}, \mathcal{M}) = \int p(\boldsymbol{\vartheta}_i \,|\, \boldsymbol{\psi}, \vec{\boldsymbol{d}}, \mathcal{M})\, p(\boldsymbol{\psi} \,|\, \vec{\boldsymbol{d}}, \mathcal{M})\, d\boldsymbol{\psi}\,. \tag{S4}$$

The dependency assumptions from Fig. S1 allow to simplify: $p(\boldsymbol{\vartheta}_i \,|\, \boldsymbol{\psi}, \vec{\boldsymbol{d}}, \mathcal{M}) = p(\boldsymbol{\vartheta}_i \,|\, \boldsymbol{\psi}, \boldsymbol{d}_i, \mathcal{M})$, and equation (S4) can be rewritten using the Bayes' theorem:

$$p(\boldsymbol{\vartheta}_i \,|\, \vec{\boldsymbol{d}}, \mathcal{M}) = \int \frac{p(\boldsymbol{d}_i \,|\, \boldsymbol{\vartheta}_i, \boldsymbol{\psi}, \mathcal{M})\, p(\boldsymbol{\vartheta}_i \,|\, \boldsymbol{\psi}, \mathcal{M})}{p(\boldsymbol{d}_i \,|\, \boldsymbol{\psi}, \mathcal{M})}\, p(\boldsymbol{\psi} \,|\, \vec{\boldsymbol{d}}, \mathcal{M})\, d\boldsymbol{\psi}\,. \tag{S5}$$

Since $p(\boldsymbol{d}_i \,|\, \boldsymbol{\vartheta}_i, \boldsymbol{\psi}, \mathcal{M}) = p(\boldsymbol{d}_i \,|\, \boldsymbol{\vartheta}_i, \mathcal{M})$, equation (S5) simplifies to

$$p(\boldsymbol{\vartheta}_i \,|\, \vec{\boldsymbol{d}}, \mathcal{M}) = \quad p(\boldsymbol{d}_i \,|\, \boldsymbol{\vartheta}_i, \mathcal{M}) \int \frac{p(\boldsymbol{\vartheta}_i \,|\, \boldsymbol{\psi}, \mathcal{M})}{p(\boldsymbol{d}_i \,|\, \boldsymbol{\psi}, \mathcal{M})}\, p(\boldsymbol{\psi} \,|\, \vec{\boldsymbol{d}}, \mathcal{M})\, d\boldsymbol{\psi}\,. \tag{S6}$$

Finally, the posterior distribution (S4) can be approximated as

$$p(\boldsymbol{\vartheta}_i \,|\, \vec{\boldsymbol{d}}, \mathcal{M}) \approx \frac{p(\boldsymbol{d}_i \,|\, \boldsymbol{\vartheta}_i, \mathcal{M})}{N_s} \sum_{k=1}^{N_s} \frac{p(\boldsymbol{\vartheta}_i \,|\, \boldsymbol{\psi}^{(k)}, \mathcal{M})}{p(\boldsymbol{d}_i \,|\, \boldsymbol{\psi}^{(k)}, \mathcal{M})}\,, \tag{S7}$$

where, $\boldsymbol{\psi}^{(k)} \sim p(\boldsymbol{\psi} \,|\, \vec{\boldsymbol{d}}, \mathcal{M})$ and N_s is sufficiently large. Thus, in order to obtain $\boldsymbol{\vartheta}_i$ samples, we first have to sample the probability distribution $p(\boldsymbol{\psi} \,|\, \vec{\boldsymbol{d}}, \mathcal{M})$, which, according to Bayes' theorem, is equal to

$$p(\boldsymbol{\psi} \,|\, \vec{\boldsymbol{d}}, \mathcal{M}) = \frac{p(\vec{\boldsymbol{d}} \,|\, \boldsymbol{\psi}, \mathcal{M})\, p(\boldsymbol{\psi} \,|\, \mathcal{M})}{p(\vec{\boldsymbol{d}} \,|\, \mathcal{M})}\,, \tag{S8}$$

where $p(\boldsymbol{\psi} \,|\, \mathcal{M})$ is the prior PDF on $\boldsymbol{\psi}$ and $p(\vec{\boldsymbol{d}} \,|\, \mathcal{M})$ is the normalizing constant. Exploiting the dependency assumption of Fig. S1 we see that

$$p(\vec{\boldsymbol{d}} \,|\, \boldsymbol{\psi}, \mathcal{M}) = \prod_{i=1}^{N} p(\boldsymbol{d}_i \,|\, \boldsymbol{\psi}, \mathcal{M}) \,, \tag{S9}$$

and the likelihood of i-th dataset can be expressed according to the total probability theorem as

$$p(\boldsymbol{d}_i \,|\, \boldsymbol{\psi}, \mathcal{M}) = \int p(\boldsymbol{d}_i \,|\, \boldsymbol{\vartheta}_i, \mathcal{M})\, p(\boldsymbol{\vartheta}_i \,|\, \boldsymbol{\psi}, \mathcal{M})\, d\boldsymbol{\vartheta}_i \,. \tag{S10}$$

Here we introduce the model $\mathcal{M}_i$ described in Fig. S1. The posterior distribution of this model will be used as instrumental density for important sampling. Under the modeling assumption $p(\boldsymbol{d}_i \,|\, \boldsymbol{\vartheta}_i, \mathcal{M}) = p(\boldsymbol{d}_i \,|\, \boldsymbol{\vartheta}_i, \mathcal{M}_i)$ (see [9]) and the use of the Bayes' theorem, equation (S10) is written as

$$p(\boldsymbol{d}_i \,|\, \boldsymbol{\psi}, \mathcal{M}) = \int \frac{p(\boldsymbol{\vartheta}_i \,|\, \boldsymbol{d}_i, \mathcal{M}_i)\, p(\boldsymbol{d}_i \,|\, \mathcal{M}_i)}{p(\boldsymbol{\vartheta}_i \,|\, \mathcal{M}_i)}\, p(\boldsymbol{\vartheta}_i \,|\, \boldsymbol{\psi}, \mathcal{M})\, d\boldsymbol{\vartheta}_i \,, \tag{S11}$$

or, equivalently, as

$$p(\boldsymbol{d}_i \,|\, \boldsymbol{\psi}, \mathcal{M}) = p(\boldsymbol{d}_i \,|\, \mathcal{M}_i) \int \frac{p(\boldsymbol{\vartheta}_i \,|\, \boldsymbol{\psi}, \mathcal{M})}{p(\boldsymbol{\vartheta}_i \,|\, \mathcal{M}_i)}\, p(\boldsymbol{\vartheta}_i \,|\, \boldsymbol{d}_i, \mathcal{M}_i)\, d\boldsymbol{\vartheta}_i \,. \tag{S12}$$

Finally, equation (S10) can be approximated as

$$p(\boldsymbol{d}_i \,|\, \boldsymbol{\psi}, \mathcal{M}) \approx \frac{p(\boldsymbol{d}_i \,|\, \mathcal{M}_i)}{N_s} \sum_{k=1}^{N_s} \frac{p(\boldsymbol{\vartheta}_i^{(k)} \,|\, \boldsymbol{\psi}, \mathcal{M})}{p(\boldsymbol{\vartheta}_i^{(k)} \,|\, \mathcal{M}_i)} \,, \tag{S13}$$

where $\boldsymbol{\vartheta}_i^{(k)} \sim p(\boldsymbol{\vartheta}_i \,|\, \boldsymbol{d}_i, \mathcal{M}_i)$ and N_s is sufficiently large. Note that in general N_s can be different for each data set $\boldsymbol{d}_i$. The advantage of this approach is that the likelihoods $p(\boldsymbol{d}_i \,|\, \boldsymbol{\vartheta}_i, \mathcal{M}_i)$, $i = 1, \ldots, N$, which are the most expensive part of the computations, are not re-evaluated for each $\boldsymbol{\psi}$.

S2 Information about hyper-parameter models for hierarchical inference

Inference for LJ 6-12 We assume that,

$$p(\boldsymbol{\vartheta} \,|\, \boldsymbol{\psi}, \mathcal{M}) = \prod_{j=1}^{3} p(\vartheta_j \,|\, \boldsymbol{\psi}, \mathcal{M}) \,, \tag{S14}$$

and we consider the following two models:

1) uniform: $p(\vartheta_j \,|\, \boldsymbol{\psi}, \mathcal{M}) = \mathcal{U}(\vartheta_j \,|\, \psi_{2j-1}, \psi_{2j-1} + \psi_{2j})$, where $\mathcal{U}(\cdot|a, b)$ is the uniform distribution with parameters a, b and $\mathcal{M}$ is set to $\mathcal{U}$,

2) log-normal: $p(\vartheta_j \,|\, \boldsymbol{\psi}, \mathcal{M}) = \mathcal{L}(\vartheta_j \,|\, \psi_{2j-1}, \psi_{2j})$, where $\mathcal{L}(\cdot|a, b)$ is the log-normal distribution of ξ with parameters a, b and $\mathcal{M}$ is set to $\mathcal{L}$.

The prior distribution on the hyper-parameters is modeled as independent uniform,

$$p(\boldsymbol{\psi} \,|\, \mathcal{M}) = \prod_{j=1}^{6} \mathcal{U}(\psi_j \,|\, a_j^{\mathcal{M}}, b_j^{\mathcal{M}}) \,, \tag{S15}$$

where $\mathcal{M} \in \{\mathcal{U}, \mathcal{L}\}$ and the constants $a_j^{\mathcal{M}}, b_j^{\mathcal{M}}$ are given in Table S1, along with the values of the log-evidences for the two models. The model $\mathcal{U}$ is according to the Bayesian model selection criterion, an order of magnitude more plausible and thus will be used for the further inference.

Table S1: $HB_{12,R}$ inference: lower bound and width for each of the hyper-parameters defined in equation (S15). Log-evidences for each hyper-prior model at the last row.

	$\mathcal{M}=\mathcal{U}$	$\mathcal{M}=\mathcal{L}$
$[a_1^{\mathcal{M}}, b_1^{\mathcal{M}}]$	[0.0, 3.0]	[-1.000, 2.0]
$[a_2^{\mathcal{M}}, b_2^{\mathcal{M}}]$	[0.0, 7.0]	[0.001, 2.5]
$[a_3^{\mathcal{M}}, b_3^{\mathcal{M}}]$	[3.0, 3.4]	[-3.000, 0.5]
$[a_4^{\mathcal{M}}, b_4^{\mathcal{M}}]$	[0.0, 2.0]	[0.001, 4.0]
$[a_5^{\mathcal{M}}, b_5^{\mathcal{M}}]$	[0.0, 0.2]	[-3.500, 0.5]
$[a_6^{\mathcal{M}}, b_6^{\mathcal{M}}]$	[0.0, 1.0]	[0.001, 2.5]
Log-ev.	-19.1401	-22.0198

Table S2: $HB_{p,R}$ inference: lower bound and width for each of the hyper-parameters defined in equation (S17). Log-evidences for each hyper-prior model at the last row.

	$\mathcal{M}=\mathcal{U}$	$\mathcal{M}=\mathcal{L}$	$\mathcal{M}=\mathcal{T}$
$[a_1^{\mathcal{M}}, b_1^{\mathcal{M}}]$	[0.0, 3.00]	[-1.000, 2.0]	[0.0500, 10.0]
$[a_2^{\mathcal{M}}, b_2^{\mathcal{M}}]$	[0.0, 7.00]	[0.001, 2.5]	[0.0010, 3.30]
$[a_3^{\mathcal{M}}, b_3^{\mathcal{M}}]$	[3.0, 3.40]	[-3.000, 0.5]	[3.0000, 4.00]
$[a_4^{\mathcal{M}}, b_4^{\mathcal{M}}]$	[0.0, 2.00]	[0.001, 4.0]	[0.0010, 0.30]
$[a_5^{\mathcal{M}}, b_5^{\mathcal{M}}]$	[6.0, 7.00]	[-2.500, 1.5]	[6.0000, 12.0]
$[a_6^{\mathcal{M}}, b_6^{\mathcal{M}}]$	[0.0, 10.0]	[0.001, 2.5]	[0.0010, 2.00]
$[a_7^{\mathcal{M}}, b_7^{\mathcal{M}}]$	[0.0, 0.20]	[-3.500, 0.5]	[0.0001, 1.00]
$[a_8^{\mathcal{M}}, b_8^{\mathcal{M}}]$	[0.0, 1.00]	[0.001, 2.5]	[0.0010, 0.30]
Log-ev.	-24.0202	-27.3889	-25.3927

Inference for LJ 6-p We assume

$$p(\boldsymbol{\vartheta} \mid \boldsymbol{\psi}, \mathcal{M}) = \prod_{j=1}^{4} p(\vartheta_j \mid \boldsymbol{\psi}, \mathcal{M}) \,, \tag{S16}$$

and consider the following three models:

1) uniform: $p(\vartheta_j \mid \boldsymbol{\psi}, \mathcal{U}) = \mathcal{U}(\vartheta_j \mid \psi_{2j-1}, \psi_{2j-1} + \psi_{2j})$ and $\mathcal{M}$ is set to $\mathcal{U}$,
2) log-normal: $p(\vartheta_j \mid \boldsymbol{\psi}, \mathcal{M}) = \mathcal{L}(\vartheta_j \mid \psi_{2j-1}, \psi_{2j})$ and $\mathcal{M}$ is set to $\mathcal{L}$,
3) truncated normal: $p(\vartheta_j \mid \boldsymbol{\psi}, \mathcal{M}) = \mathcal{T}(\vartheta_j \mid \psi_{2j-1}, \psi_{2j})$, where $\mathcal{T}(\cdot|a, b)$ is the truncated normal distribution with parameters a, b and $\mathcal{M}$ is set to $\mathcal{T}$.

The prior distribution on the hyper-parameters is modeled as independent uniform,

$$p(\boldsymbol{\psi} \mid \mathcal{M}) = \prod_{j=1}^{8} p(\psi_j \mid \mathcal{M}) = \prod_{j=1}^{8} \mathcal{U}(\psi_j \mid a_j^{\mathcal{M}}, b_j^{\mathcal{M}}) \,, \tag{S17}$$

where $\mathcal{M} \in \{\mathcal{U}, \mathcal{L}, \mathcal{T}\}$ and the constants $a_j^{\mathcal{M}}, b_j^{\mathcal{M}}$ are given in Table S2, along with the values of the log-evidences for the three models. As it can be seen, the uniform model is the most plausible one.

S3 Posterior parameter distribution for LJ 6-12 and LJ 6-p

This section presents the posterior distributions for LJ 6-12 and LJ 6-p potentials obtained in HB TMCMC runs for each thermodynamic condition, as well as, the distribution for the quantum dimer-based Bayesian inference. Each plot contains all the TMCMC samples of the last stage and it consists of the following parts: histograms of marginal distributions of parameters are shown on the diagonal, projections of the samples to all pair 2-d subspaces in the parameter space colored by the log-likelihood values are given above the diagonal, the corresponding densities constructed via a bivariate kernel estimate are depicted below the diagonal. Green star shows the parameters from Ref. [10], green square indicates the parameters from Ref. [11], and green circle marks the parameters from Ref. [12, 13].

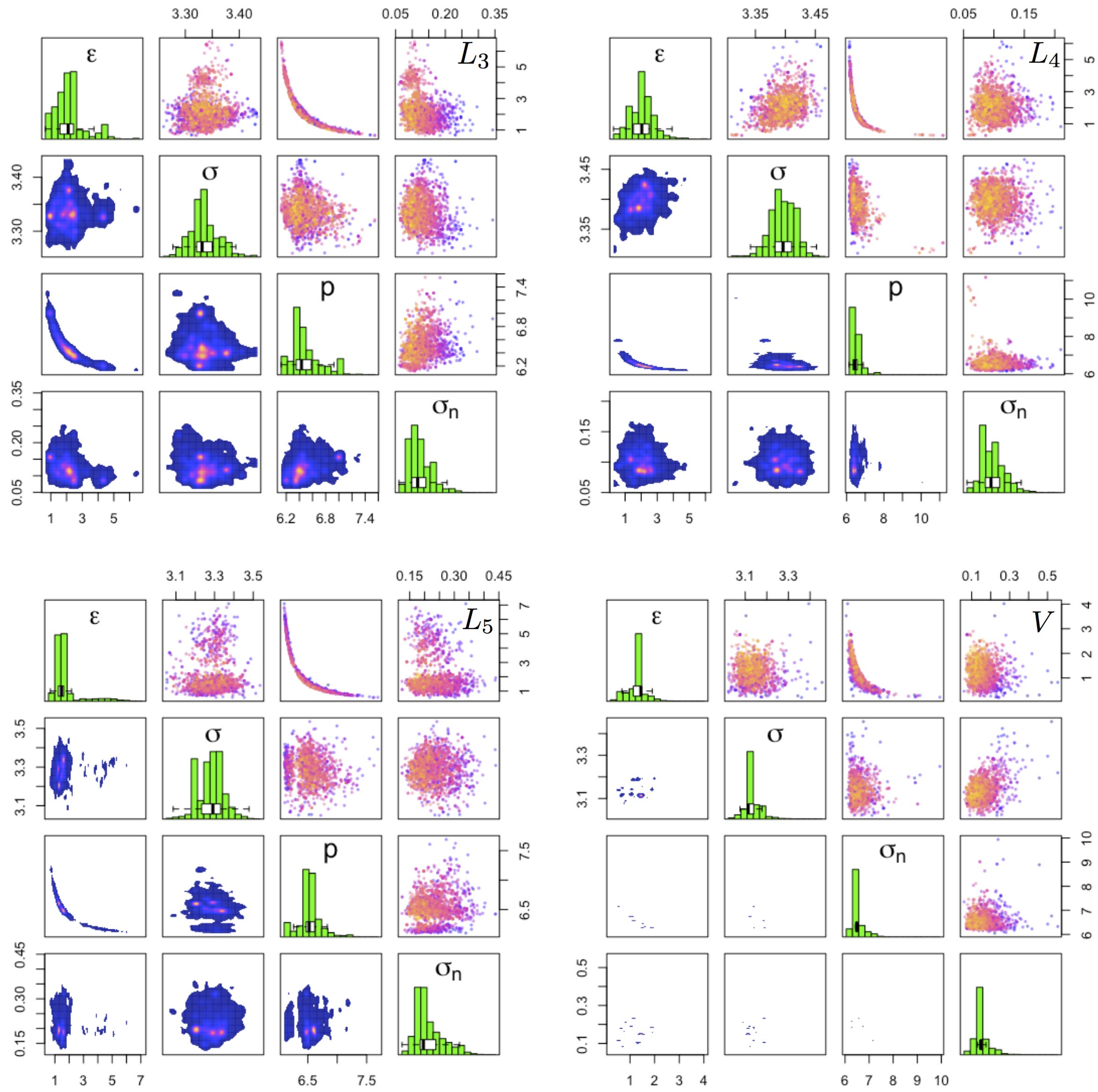

Figure S2: LJ parameters distributions obtained in $HB_{p,R}$ for L_3 (top left), L_4 (top right), L_5 (bottom left) and V (bottom right).

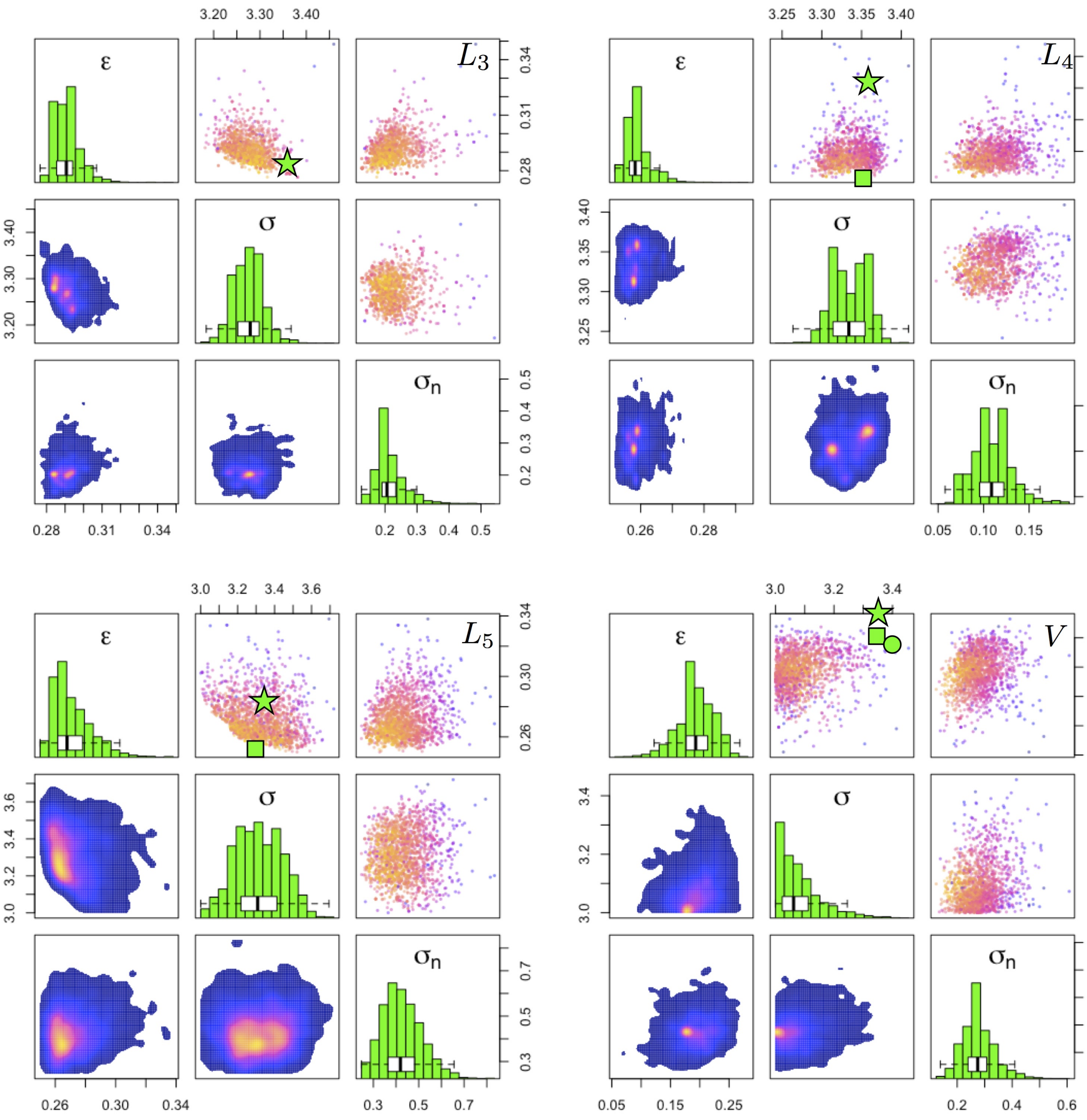

Figure S3: LJ parameters distributions obtained in $HB_{12,R}$ for L_3 (top left), L_4 (top right), L_5 (bottom left) and V (bottom right).

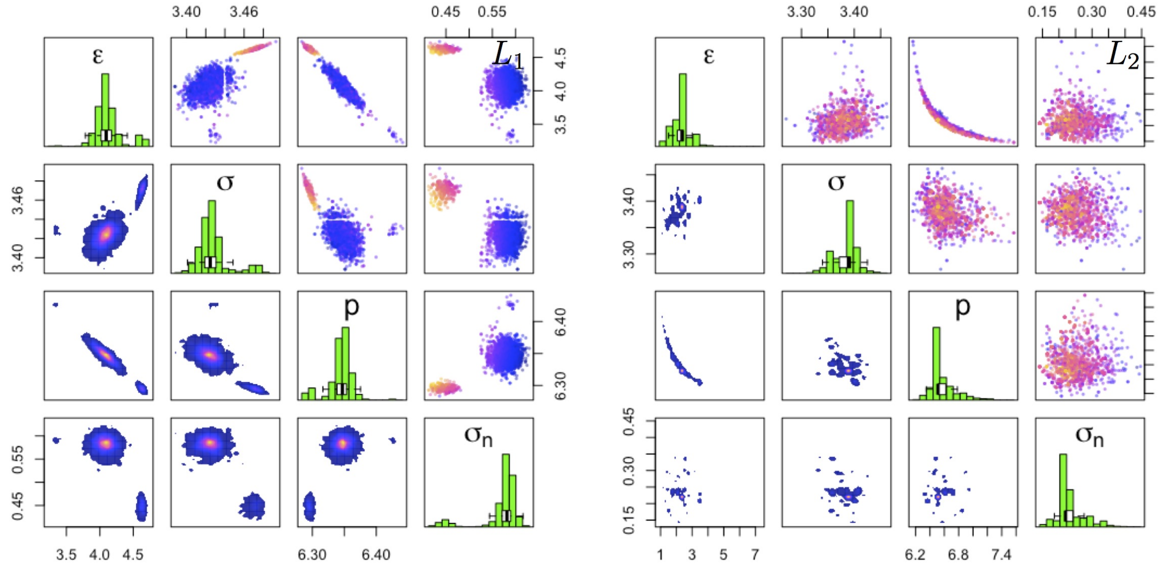

Figure S4: LJ parameters distributions obtained in $HB_{p,R}$ for L_1 (left) and L_2 (right).

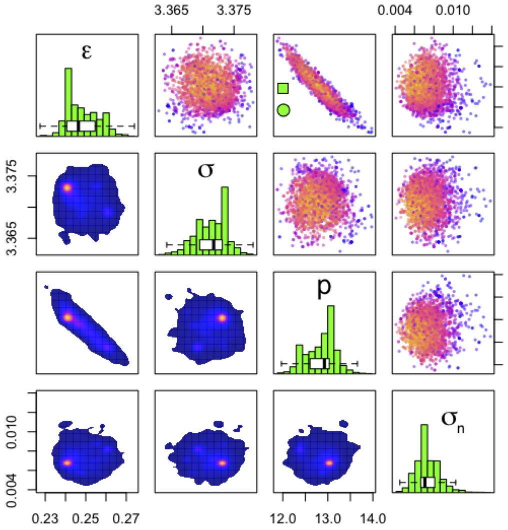

Figure S5: LJ parameters distributions obtained in $B_{p,Q}$.

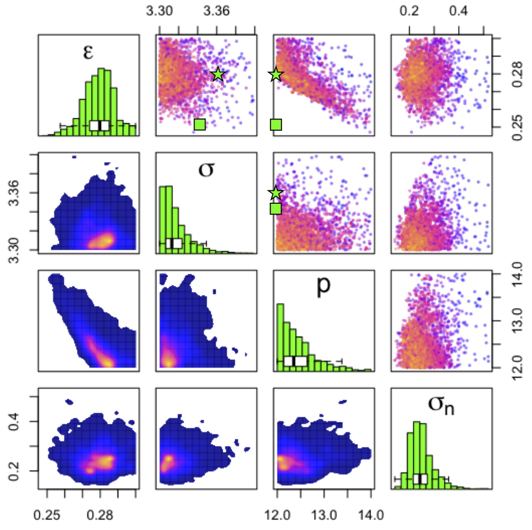

Figure S6: LJ parameters distributions obtained in $B_{p,R}$ for L_3 with the prior for p restricted to $[12, 14]$.

References

[1] W. K. Hastings. Monte Carlo sampling methods using Markov chains and their applications. *Biometrika*, 57(1):97–109, 1970.

[2] W. R. Gilks. *Markov chain Monte Carlo.* John Wiley and Sons, Ltd., 2005.

[3] D. Gamerman and H. F. Lopes. *Markov chain Monte Carlo: stochastic simulation for Bayesian inference.* CRC Press, 2006.

[4] C. J. Geyer. Practical Markov chain Monte Carlo. *Statistical Science*, pages 473–483, 1992.

[5] P. H. Peskun. Optimum Monte Carlo sampling using Markov chains. *Biometrika*, 60(3):607–612, 1973.

[6] J. Ching and Y. Chen. Transitional Markov chain Monte Carlo method for Bayesian model updating, model class selection, and model averaging. *J. Eng. Mech.*, 133(7):816–832, 2007.

[7] P. Angelikopoulos, C. Papadimitriou, and P. Koumoutsakos. Bayesian uncertainty quantification and propagation in molecular dynamics simulations: A high performance computing framework. *J. Chem. Phys.*, 137:144103, 2012.

[8] P. E. Hadjidoukas, P. Angelikopoulos, C. Papadimitriou, and P. Koumoutsakos. Pi4U: A high performance computing framework for Bayesian uncertainty quantification of complex models. *J. Comp. Phys.*, 284:1–21, 2015.

[9] S. Wu, P. Angelikopoulos, G. Tauriello, C. Papadimitriou, and P. Koumoutsakos. Fusing heterogeneous data for the calibration of molecular dynamics force fields using hierarchical Bayesian models. *J. Chem. Phys.*, 145:244112, 2016.

[10] J. A. Barker, R. A. Fisher, and R. O. Watts. Liquid argon: Monte Carlo and molecular dynamics calculations. *Mol. Phys.*, 21:657–673, 1971.

[11] John A. White. Lennard-Jones as a model for argon and test of extended renormalization group calculations. *J. Chem. Phys.*, 111:9352–9356, 1999.

[12] A. Rahman. Correlations in the motion of atoms in liquid argon. *Phys. Rev.*, 136:A405–A411, 1964.

[13] L. A. Rowley, D. Nicholson, and N. G. Parsonage. Monte Carlo grand canonical ensemble calculation in a gas-liquid transition region for 12-6 argon. *J. Comp. Phys.*, 17:401–414, 1975.
